# A Comparative Study of Solifenacin, Mirabegron, and Their Combination as Bladder Relaxants in the Management of Overactive Bladder

**DOI:** 10.7759/cureus.45612

**Published:** 2023-09-20

**Authors:** Shailendra Kumar, Vidushi Tiwari, Dileep K Chaurasia, Sudheer Kumar, Shirish Mishra

**Affiliations:** 1 Surgery, Maharshi Vashishtha Autonomous State Medical College, Basti, IND; 2 Surgery, Motilal Nehru Medical College, Prayagraj, IND; 3 Urology, Motilal Nehru Medical College, Prayagraj, IND

**Keywords:** efficacy, overactive bladder, solifenacin, mirabegron, combination therapy

## Abstract

Introduction

Overactive bladder (OAB) is a medical state that presents as the urgency of urine and increased frequency of micturition and is diagnosed on the basis of the presence of these symptoms in the absence of other explainable diagnoses. The management of this condition includes conservative management, medical management/pharmacotherapy, and surgical management. The overactive bladder has been treated with smooth muscle relaxants, but there are conflicting results. Hence, this study aimed to assess the result of the two smooth muscle relaxants, mirabegron and solifenacin, and their combination to manage an overactive bladder.

Methodology

A clinical trial was conducted at Swaroop Rani Nehru Hospital, Motilal Nehru Medical College, Prayagraj, India, over the period from November 2019 to December 2020. Ninety patients with OAB were divided into three groups: G1, G2, and G3. These groups were administered solifenacin, mirabegron, and a combination of mirabegron and solifenacin (S+M), respectively. Follow-ups were conducted at 2, 4, 12, and 18 weeks for evaluation. Data were entered into IBM SPSS Statistics for Windows, Version 23 (Released 2015; IBM Corp., Armonk, New York, United States). Appropriate statistical tests, including the chi-square and ANOVA, were employed in this study.

Observation

The combination of mirabegron and solifenacin was significantly more effective in terms of response compared to solifenacin alone. There was no significant difference between solifenacin versus mirabegron, or between mirabegron (M) and the combination of mirabegron (M) and solifenacin (S). Side effects were more severe in patients taking high doses of solifenacin.

Conclusion

The S + M combination has higher efficacy than solifenacin and mirabegron when given alone.

## Introduction

Overactive bladder (OAB) is a medical state that presents as urgency of urine and increased micturition frequency which may be associated with urge incontinence and nocturia. However, there must be an absence of local pathologies or metabolic factors that might produce these symptoms. The causative factors include uroepithelial factors, neurogenic factors damaging inhibitory pathways, myogenic factors, and miscellaneous factors such as infections like recurrent urinary tract infection (UTI), cystitis, chronic prostatitis, etc., as well as metabolic syndrome and bladder outlet obstruction resulting from prostate cancer, benign prostatic hyperplasia, bladder cancer, urethral stricture, and bladder calculi [1].

Risk factors include advanced age, post-menopausal women, pelvic organ prolapse, incontinence surgery, neurological diseases, depression, hypertension, recurrent UTI, prostatitis, irritable bowel syndrome, sleep apnea, diet factors like caffeine, artificial sweeteners, carbonated drinks, spicy food, etc., smoking and alcohol intake, and both excessive and inadequate fluid intake [2].

It is diagnosed based on the presence or absence of symptoms of the lower urinary tract (LUTS) in the absence of other explainable diagnoses. Management of OAB can be conservative (lifestyle changes, control techniques), medical/pharmacological (Antimuscarinics, Alpha agonists, Antidepressants, Cyclo-oxygenase inhibitors, Beta-3 agonists, etc.), or surgical (cystoplasty, detrusor myectomy, neural modulation/stimulation). When all management fails, the following alternatives are used: Suprapubic or urethral catheters, urethral closure and diversions, appliances, etc. [3].

The use of smooth muscle relaxants for treating overactive bladders has been introduced recently, but the results are conflicting [1]. Hence, this study aimed to assess the effect of the two smooth muscle relaxants, Mirabegron and solifenacin, and their combined effect on the treatment of overactive bladder.

## Materials and methods

A randomized clinical trial was done in Swaroop Rani Nehru Hospital of Motilal Nehru Medical College, Prayagraj, India, from November 2019 to December 2020 among 90 patients of OAB.

Inclusion criteria

All newly diagnosed patients of any age, complaining of increased urination frequency, urinary urgency, urinary incontinence, and nocturia were included in the study.

Exclusion criteria

Patients who did not give consent, those with the presence of any pathology (liver disease, closed-angle glaucoma, stress incontinence, neurological cause of abnormal detrusor activity), patients in which drugs could not be given (hypersensitivity to drugs, any history of gastric retention, closed-angle glaucoma), and patients who had a history of any operation of the urinary bladder, urethra, or endoscopic intervention were excluded from the study.

Sampling procedure

The institute's Ethics Committee clearance was obtained before the initiation of the study (approval no. ECR/922/INST/UP/2017). All new patients were consecutively randomized and divided into three groups: G1, G2, and G3. Ninety patients were evaluated for the entire study period, that is, 30 patients in each group. The groups were allotted the drug as follows: (1) G1-Solifenacin (S) (doses-5mg, 10mg); (2) G2-Mirabegron (M) (doses25mg, 50 mg); and (3) G3-Combination of S+M (S-5mg +M-25mg)/(S-5mg+M-50mg).

The patients were examined and evaluated. A semi-structured questionnaire was employed for data collection from patients. Follow-ups were carried out at 2, 4, 12, and 18 weeks, with no patients lost to follow-up. At every follow-up, the following points were evaluated: micturition frequency per 24 hours, urgency episodes per 24 hours, incontinence episodes per 24 hours, urge incontinence episodes per 24 hours, and nocturia episodes per 24 hours.

The data were entered into IBM SPSS Statistics for Windows, Version 23 (Released 2015; IBM Corp., Armonk, New York, United States). Appropriate statistical tests including chi-square, ANOVA, and repeated measures ANOVA were utilized in this study. In this study, a comparison of improvement and side effects was measured in terms of Good, Bad, or No response.

Good Response

This was assigned to patients if there was a decrease in the number of nocturia episodes, decreased urinary frequency, fewer episodes of urinary incontinence, improvements in lifestyle, and a decrease in urge incontinence.

Bad Response

Patients were assigned this if there was no decrease or an increase in nocturia episodes, no decrease or an increase in urinary frequency, no decrease or an increase in incontinence episodes, no improvement in lifestyle, and no decrease or an increase in urge incontinence.

No Response

After prolonged treatment, patients were assigned to this group if their response was between Good and Bad, or if they showed no response or improved symptoms.

## Results

In this study of 90 patients presenting with symptoms of overactive bladder, the efficacy of solifenacin, mirabegron, and their combination was compared over a one-year period from December 2019 to November 2020. Patients were allocated to groups 1 through 3, and treatments were administered using solifenacin, mirabegron, and a combination of the two, respectively.

**Figure 1:**
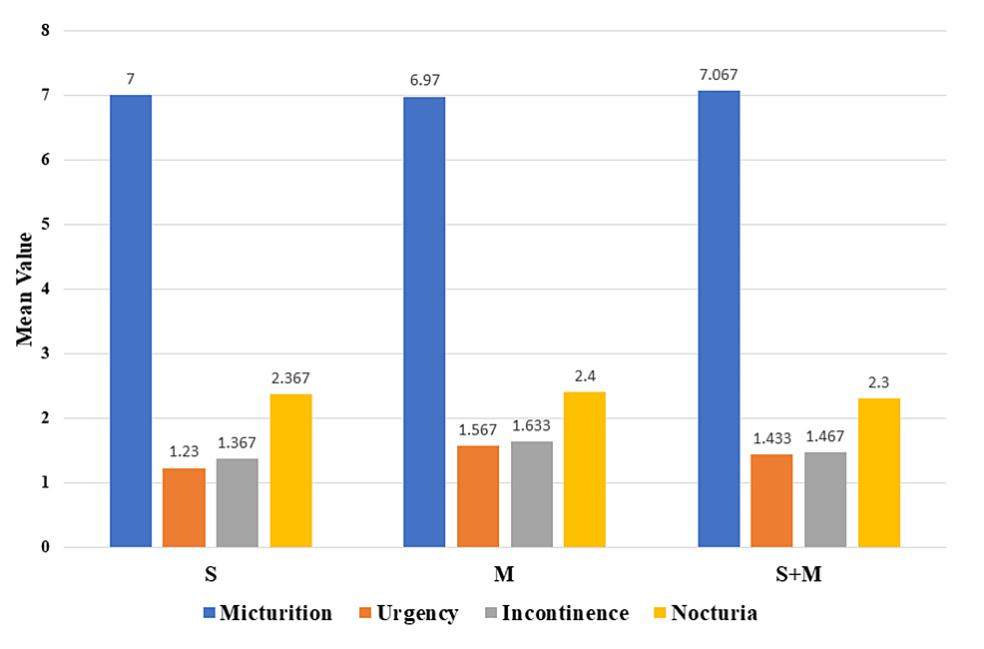
Baseline value of symptoms of patients S: solifenacin; M: mirabegron; S+M: solifenacin + mirabegron.

The study showed a male preponderance and the mean age of the participants was approximately 60 years across all three groups. Among all the groups, the highest mean baseline score for micturition episodes per 24 hours and mean urge incontinence score were observed in group 3, with scores of 7.067±0.868 and 1.467±0.630, respectively. The highest mean baseline score for urgency episodes per 24 hours and mean incontinence score were noted in group 2, with scores of 1.566±0.820 and 1.630±0.850, respectively (Figure 1, Table 1).

**Table 1:** Distribution based on clinicosocial characteristics *n (percentage). S: solifenacin; M: mirabegron; S+M: solifenacin + mirabegron; SD: standard deviation.

Variables		Groups		
		Solifenacin (S) (N=30)	Mirabegron (M) (N=30)	S+M (N=30)
Gender	Male	25 (83.33%)	28 (93.33%)	26 (86.66%)
	Female	5 (16.66%)	2 (6.66%)	4 (13.33%)
Age (years)*	Mean age	61.5	61.76	59.33
Symptoms (mean ± SD)	Micturition	7.000±0.871	6.970±0.760	7.067±0.868
	Urgency	1.233±0.727	1.566±0.820	1.433±0.630
	Incontinence	1.367±0.720	1.630±0.850	1.467±0.630
	Urge incontinence	1.167±0.746	1.400±0.770	1.467±0.630
	Nocturia	2.367±0.490	2.400±0.563	2.400±0.651

**Table 2:** Efficacy regarding decrease in micturition episodes/24 hours S: solifenacin; M: mirabegron; S+M: solifenacin + mirabegron; p: probability of chance error.

Groups	Duration of follow-up					p-value
	Baseline	2 weeks	4 weeks	12 weeks	18 weeks	
Micturition episodes/24 hours						
S	7±0.871	6.133±0.776	5.1±0.712	4.2±0.610	3.53±0.51	<0.001
M	6.97±0.76	6±0.742	5±0.74	4±0.74	3.3±0.47	<0.001
S+M	7.067±0.868	6.067±0.868	5.033±0.85	3.733±0.583	3.067±0.25	<0.001
Urgency episode/24 hours						
S	1.23±0.727	0.733±0.520	0.433±0.504	0.233±0.430	0.2±0.406	<0.001
M	1.567±0.8172	0.967±0.556	0.6±0.563	0.3±0.466	0.133±0.346	<0.001
S+M	1.433±0.626	0.933±0.365	0.367±0.490	0.133±0.345	0.1±0.305	<0.001
Incontinence episode/24 hours						
S	1.367±0.718	0.933±0.449	0.533±0.507	0.2±0.406	0.167±0.379	<0.001
M	1.633±0.850	0.9±0.607	0.533±0.507	0.233±0.430	0.1±0.305	<0.001
S+M	1.467±0.628	0.933±0.365	0.367±0.490	0.133±0.345	0.133±0.345	<0.001
Nocturia episodes/24 hours						
S	2.367±0.490	1.367±0.490	0.367±0.49	0.367±0.49	0.367±0.49	1.000
M	2.4±0.563	1.433±0.504	0.233±0.4302	0.23±0.430	0.23±0.43	<0.001
S+M	2.3±0.651	1.367±0.490	0.1±0.315	0.067±0.254	0.033±0.183	<0.001

The table indicates that the combination of solifenacin (S) and mirabegron (M) is notably more effective than using either of these medications on their own. The combination of S+M led to a mean reduction of 4 episodes of micturition per 24 hours. This compares favorably to a reduction of 3.47 episodes for the Solifenacin group and 3.67 episodes in the Mirabegron group. Similarly, mean urgency episodes decreased by 1.33 per 24 hours when treated with the S+M combination.

Moreover, the combination of solifenacin and mirabegron appears equivalent to mirabegron alone and superior to solifenacin in decreasing the number of incontinence episodes per 24 hours. Specifically, the S+M combination led to a mean decrease of 1.34 incontinence episodes per 24 hours, compared to 1.2 for the solifenacin group and 1.4 for the mirabegron group.

In terms of nocturia episodes per 24 hours, the S+M combination was superior to either of the individual drugs, reducing episodes by 2.267 per 24 hours. This was better than the 1.977 episodes reduced in the Solifenacin group and 2.17 episodes in the Mirabegron group (Table 2).

**Table 3:** Comparison of response of drugs Values in n(%). S: solifenacin; M: mirabegron; S+M: solifenacin + mirabegron.

Drugs/response	Good	Bad/no response
Solifenacin (S)	19(63.33%)	11(36.66%)
Mirabegrone (M)	23(76.66%)	7(23.33%)
S+M	26(86.66%)	4(13.33%)

The majority of the study participants in the S+M group (86.66%) showed good results with the drug, compared with those in the mirabegron (76.66%) and solifenacin (63.33%) groups (Table 3).

**Table 4:** Comparison of response of drugs in three groups* Values in n(%). *chi-square test. S: solifenacin; M: mirabegron; S+M: solifenacin + mirabegron; p: probability of chance error.

Groups	Good	No/bad response	P value
Solifenacin vs mirabegron			
Group 1 (S)	19 (63.3)	11 (36.6)	0.2698
Group 2 (M)	23 (76.6)	7 (23.3)	
Solifenacin vs mirabegron (M) + solifenacin (S)			
Group 1 (S)	19 (63.3)	11 (36.6)	0.0368
Group 3 (M+S)	26 (86.7)	4 (13.3)	
Mirabegron (M) vs mirabegron (M)+solifenacin (S)			
Group 2 (M)	23 (76.6)	7 (23.3)	0.317
Group 3 (M+S)	26 (86.7)	4 (13.3)	

The table shows that the combination of mirabegrone + solifenacin was significantly superior in terms of response than solifenacin. There was no significant difference between solifenacin vs mirabegron and mirabegron (M) vs mirabegron (M)+solifenacin(S) (Table 4).

**Table 5:** Distribution based on side effects of the groups Values in n(%). S: solifenacin; M: mirabegron; S+M: solifenacin + mirabegron.

Side effect	Acute retention of urine	Headache	Acute exacerbation of hypertension	Anticholinergic
S	2 (6.66)	3 (10.00)	2 (6.66)	4 (13.33)
M	2 (6.66)	2 (6.66)	2 (6.66)	2 (6.66)
S+M	2 (6.66)	1 (3.33)	1 (3.33)	3 (10.00)

**Figure 2:**
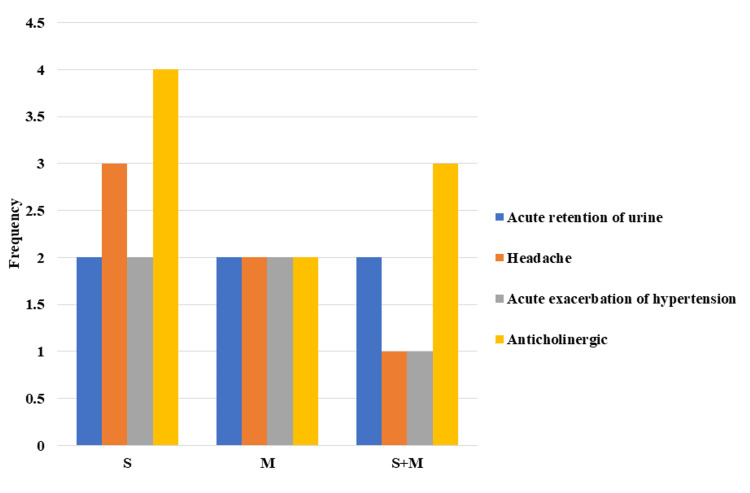
Comparing side effects of drugs S: solifenacin; M: mirabegron; S+M: solifenacin + mirabegron.

Analysis of the side effects data for the three drugs reveals that the most commonly reported adverse events were anticholinergic in nature. These adverse events ranged from mild to moderate symptoms, and no patients discontinued the medication due to side effects. However, it is worth noting that side effects were more severe in patients who were taking higher doses of solifenacin (Table 5, Figure 2).

## Discussion

Overactive bladder is a prevalent issue in elderly patients, and antimuscarinics are currently the most commonly prescribed medications for treatment. Mirabegron, a newly FDA-approved drug, offers an additional option for treating overactive bladder. However, solifenacin and other antimuscarinics remain widely used and are the mainstay of treatment. In this comparative study, we evaluated the clinical efficacy of flexible dosing of solifenacin (5mg and 10mg), mirabegron (25mg and 50mg), and their combination (solifenacin 5mg + mirabegron 25mg/50mg). The study involved 90 patients, and there were no losses to follow-up.

Demographic characteristics of the study participants

The study showed a male preponderance, and the mean age of the participants across all three groups was approximately 60 years. These characteristics are comparable to those found in a study by Tang et al., which also compared solifenacin and a combination of solifenacin and mirabegron. In that study, the mean patient age was 45 years, and there were no discernible differences between the two groups in terms of age and gender [4].

Efficacy of the drug

In this study, almost all episodes of incontinence were episodes of urge incontinence. This study demonstrated a statistically significant reduction in all symptoms of overactive bladder syndrome across all three groups. Changes were measured by a decrease in pad use and the wetting of clothes, as well as in episodes of nocturia. These changes were observed after a duration of 18 weeks. A combination of mirabegron and solifenacin was found to have superior efficacy and response in reducing the symptoms of overactive bladder compared to either solifenacin or mirabegron alone.

Our study corroborated the findings of previous studies conducted on the same subject. For example, Sacco et al. reported that the incidence and severity of treatment outcomes were similar to those observed with antimuscarinics [5]. Maman et al. found comparable efficacy between mirabegron 50 mg and other antimuscarinics in improving micturition frequency, urgency, and incontinence, however, they noted that solifenacin 10 mg was more efficacious than mirabegron 50 mg [6]. Batista et al. revealed that both treatments led to a clinically meaningful reduction in symptoms [7]. Abrams et al. stated that combination therapy with mirabegron and solifenacin improved voiding volume, urgency, and micturition frequency more effectively than monotherapy with 5 mg of solifenacin [8]. Drake et al. observed that the combination treatment was superior to solifenacin monotherapy in reducing symptoms of incontinence and urinary frequency [9]. Jayarajan et al. examined newer treatment options for overactive bladder, focusing on their pharmacology, effectiveness, side-effect profile, tolerability, and impact on patient quality of life [10]. The majority of anticholinergic medications were found to have comparable efficacies [11]. Bragg et al. found that mirabegron effectively reduced the average number of urination and incontinence episodes per day [11]. Homma et al. noted significant improvements in efficacy outcomes from baseline to the end of therapy in all groups receiving either solifenacin or mirabegron [12]. Xu Y et al. demonstrated that combination therapy was more effective than solifenacin alone [13]. Similar to our study, they found that combination therapy was better at controlling overactive bladder symptoms such as micturition frequency, urgency, urinary incontinence, and nocturia [13].

Adverse events associated with the drugs

In the present study, the analysis of side effects for three drugs shows that the most commonly reported side effects were anticholinergic side effects. The adverse effects were mainly mild to moderate and none of the patients discontinued the drug because of side effects. However, side effects were more severe in patients taking high doses of solifenacin.

Sacco et al. reported that serious adverse events were comparable to antimuscarinics, but with a significantly lower incidence of dry mouth as compared with antimuscarinics [5]. According to Khullar et al., the incidence of adverse effects was similar among all the treatment groups [14]. Nitti et al. showed that mirabegron was tolerated well and showed an acceptable safety margin with commonly reported adverse events (≥ 3%) was nasopharyngitis, dose-related hypertension, and dose-related urinary tract infection (UTI) [15]. Batista et al. revealed that both treatments were acceptable, with a minimum incidence of dry mouth in the mirabegron group [7]. Abrams et al., show that combination therapy (S+M) was tolerated well, compared to monotherapy or a placebo, with no significant extra safety findings [8]. Jayarajan et al. reported that the majority of anticholinergic medications have main side effects related to muscarinic receptors. For individuals who are intolerant to or receiving insufficient anticholinergic medication, newer medicines with other sites of action, such as mirabegron (Beta-3 adrenergic), offer additional treatment choices. The anticholinergic side effect was more pronounced with antimuscarinics and less pronounced with mirabegron, which also had superior toleration and quicker response in terms of quality of life [10]. Bragg et al. found that mirabegron frequently causes side effects like hypertension, nasopharyngitis, urinary tract infections, headache, constipation, upper respiratory tract infections, arthralgia, diarrhea, tachycardia, abdominal pain, and exhaustion [11]. Homma et al. showed that in patients with OAB receiving solifenacin 2.5 mg or 5 mg once daily, combination therapy with mirabegron 25 mg once daily for 16 weeks, with an optional dose increase to 50 mg at week 8, was well tolerated [12]. With mirabegron and solifenacin combination therapy, OAB symptoms significantly improved from baseline to EOT. Similarly, in our trial, combination therapy had better control of OAB symptoms and had fewer side effects than solifenacin monotherapy. Additionally, combination therapy produced results that lasted over time. In their study, Xu et al. showed that the safety evaluations, which cover frequent adverse events that develop during therapy and discontinuation owing to adverse events were less frequent with the combination therapy (solifenacin + mirabegron) [13]. Combination therapy was better tolerated than solifenacin/mirabegrone alone and also had fewer side effects.

If patients felt their treatment was ineffective after four weeks of active treatment, our study's design permitted them to request an increase in drug dose, and then patients were re-evaluated after the duration of 18 weeks. After a dose increase, a small number of individuals on solifenacin reported experiencing antimuscarinic adverse effects such as dry mouth, diarrhea, and impaired vision. However, the symptoms and complaints are less or absent with mirabegrone and its combination. Taken together, these results imply that patients may be less tolerant of lack of efficacy than of antimuscarinic side effects. Despite rising side effects, in fact, about half of the patients wanted a dose increase. All of these factors are crucial because an effective treatment should strike the ideal balance between the greatest possible improvement in clinical symptoms and accepted tolerability, leading to a discernible and valuable increase in quality of life.

The main limitation of this study was the short (18 weeks) duration of treatment. The study needs a larger group and long-term follow-up to study the side effects and neoplastic potential of mirabegron, and also for a significant increase in blood pressure and cardiovascular symptoms. In our trial, mirabegron was used to treat OAB and showed higher efficacy and tolerability with fewer side effects. Mirabegron provides a new class of medication for OAB with proven efficacy and good tolerability.

## Conclusions

The combination therapy exhibits greater efficacy than either solifenacin or mirabegron when administered individually. Mirabegron demonstrates superior efficacy to solifenacin in reducing episodes of micturition and urge incontinence per 24 hours. Both the combination therapy and mirabegron are nearly equally effective but surpass solifenacin in reducing episodes of urgency and incontinence per 24 hours. In terms of reducing nocturia episodes per 24 hours, solifenacin and mirabegron are equally effective; however, the combination therapy proves to be superior. The most frequently reported side effects across all three groups are anticholinergic in nature, yet these side effects occur significantly less often in groups treated with Mirabegron and the combination therapy. Therefore, for the treatment of overactive bladder, the combination therapy can be recommended as the first-line medication due to its enhanced control and rapid response over symptoms of OAB.
